# The Light-Intensity-Affected Aroma Components of Green Tea during Leaf Spreading

**DOI:** 10.3390/foods13152349

**Published:** 2024-07-25

**Authors:** Youyue He, Shujing Liu, Yuzhong Kang, Rajiv Periakaruppan, Jing Zhuang, Yuhua Wang, Xuan Chen, Xinqiu Liu, Xinghui Li

**Affiliations:** 1 College of Horticulture, Nanjing Agricultural University, Nanjing 210095, China; 2022204005@stu.njau.edu.cn (Y.H.); 2023085@njau.edu.cn (S.L.); 2022104079@stu.njau.edu.cn (Y.K.); zhuangjing@njau.edu.cn (J.Z.); wangyuhua@njau.edu.cn (Y.W.); chenxuan@njau.edu.cn (X.C.); 2 Department of Biotechnology, PSG College of Arts & Science, Coimbatore 641 014, India; rajivsmart15@gmail.com; 3 College of Humanities and Social Development, Nanjing Agricultural University, Nanjing 210095, China; 4 Huanghai Science and Technology Innovation Research Institute of Shandong, Rizhao 276801, China

**Keywords:** green tea, leaf spreading, light intensity, volatile substance, odor activity value, sensory quality

## Abstract

Leaf spreading is a key processing step that affects the aroma formation of green tea. The effects of a single-light wavelength on the aroma and taste of tea have been extensively studied. Less attention has been paid to the effect of different complex light intensities on the formation of green tea’s volatile aroma during leaf spreading. The current study was designed to evaluate how leaf spreading under different complex light intensities relates to the quality of green tea. Using headspace solid-phase micro-extraction and gas chromatography-mass spectrometry (HS-SPME/GC-MS), volatile flavor compounds in green tea were analyzed during leaf spreading in five different light conditions. Multivariate statistical analysis and odor activity values (OAVs) were used to classify these samples and identify key odors. Eight distinct groups, including ninety volatile compounds, were detected. The most prevalent volatile compounds found in green tea samples were hydrocarbons and alcohols, which accounted for 29% and 22% of the total volatile compounds, respectively. Fourteen volatile compounds (OAV > 1) were identified as key active differential odorants. The chestnut-like aroma in green tea was mostly derived from 3-methyl-butanal and linalool, which were significantly accumulated in medium-intensity light (ML).

## 1. Introduction

Tea, made from the commercially important plant *Camellia sinensis* (L.), is one of the world’s second most popular drinks. It has a pleasant flavor and health benefits [1]. There are many kinds of tea on the market that can be divided into green tea, black tea, oolong tea, white tea, dark tea, and yellow tea, according to the combination of sensory flavor and processing procedures of tea products [2]. Due to its acclaimed health benefits, green tea has become more popular, being consumed worldwide nowadays.

The processes of spreading, fixing, rolling, and drying are typically involved in green tea production [3]. The first and most important stage in the processing of green tea is spreading, which is crucial for creating aroma in premium green tea [4]. After spreading, a certain amount of water in the fresh leaves evaporates, the color of the fresh leaves becomes darker, the leaves become soft, and the plasticity of the tea is enhanced, all of which are conducive to the shape of the tea leaves. During spreading, photosynthesis and respiration are still ongoing and the fresh leaves are still living [5,6]. Actually, the formation and release of aromatic compounds occur during this process [7]. Therefore, the reasonable spreading of fresh leaves plays an important role in the formation of tea color, aroma, taste, and shape.

The formation of volatile compounds in the growth of tea plants is affected by biotic or abiotic impactors [8]. Among the abiotic factors, light is the key factor affecting tea flavor generation [9,10]. A study has shown that both red light and blue light can improve the content of endogenous volatiles in tea, particularly affecting volatile fatty acid derivatives (VFADs), volatile phenylpropanoids/benzenoids (VPBs), and volatile terpenes (VTs) [11]. In addition, the addition of bule/green light can promote the decomposition process of chlorophyll in tea plants, thereby increasing the content of amino acids in chlorophyll leaves, thereby further improving the umami taste of tea and achieving a higher economic value [12]. These results indicate that light intensity and wavelength could effectively regulate the formation of most tea volatiles and ultimately affect the quality of tea products.

Currently, extensive studies have proven that adding light sources during the spreading/withering process could effectively improve the quality of tea. Red light significantly increases the total amount of volatile components in the early and middle stages of the withering process. With the increase in red-light intensity, the total amount of esters significantly increases and the total amount of ketones significantly decreases [9]. Red- or blue-light treatment during withering resulted in a significant decrease in ester catechin content in black tea. Black tea treated with red light had a high aroma score, and black tea treated with blue light had a high taste score, thus affecting the quality of the finished tea [13]. In fact, treatment with monochromatic yellow, orange, and red lights during withering also could increase overall black tea quality [14]. Red-light withering (RLW) could significantly reduce the bitterness and astringency of summer–autumn black tea and improve its taste quality [15]. Red-light spreading could enrich the tea variety, content of green tea aroma substances, improve the freshness, and improve summer–autumn green tea quality [10]. In conclusion, adding external light sources to the spreading/withering process could significantly improve tea quality.

At present, the selection of light quality during tea spreading/withering has been widely studied, but the understanding of how the variation in light-intensity parameters can improve tea aroma quality is still limited. Here, different light-intensity treatments were applied to the tea spreading process for studying green tea volatile aroma compounds under different treatments. The study will provide a scientific, theoretical, and technical basis for the optimization of processing technology and processing equipment for premium green tea.

## 2. Materials and Methods

### 2.1. Fresh Tea Leaves

The fresh tea leaves of ‘Fudingdabai’, a tea plant cultivar, were used in the experiments. We plucked the tea leaves (one bud with two leaves) at Jiangsu Tea Expo Park (Nanjing, China). ‘Fudingdabai’ is a breed of clones with early spring germination, strong sprout and leaf fertility, high germination density, strong, yellow-green in color, and very fuzzy. It is suitable for producing white tea, green tea, and black tea.

### 2.2. External Light Source

**Figure 1:**
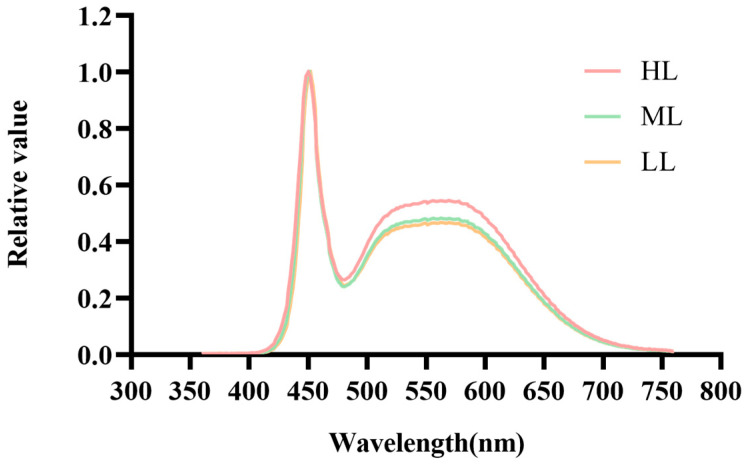
LED photon-distribution spectrum of full-spectrum composite light. Low-intensity light (LL; full-spectrum composite light, 400–750 nm; 75 μmol∙m^−2^∙s^−1^), medium-intensity light ((ML) 150 μmol∙m^−2^∙s^−1^), and high-intensity light ((HL) 300 μmol∙m^−2^∙s^−1^).

The light-emitting diode (LED) equipment (Xuanmei Automation Technology Co., Ltd., Nanjing, China) is based on our previous research [16]. The spectral parameters of the illumination device are shown in Figure 1.

### 2.3. Withering Experiment with Different Light Conditions

The treatment of freshly plucked tea leaves and the light-intensity conditions were obtained from He et al. [16]. In this study, five treatments were set up, namely high (HL)-, medium (ML)-, and low (LL)-light treatments, and indoor natural-light (NL) and dark (DARK) treatments were used as controls.

### 2.4. Tea Sample Preparation

For use in later tests, the tea samples were kept in a refrigerator at 4 °C. The spreading time, fixing, and drying conditions were obtained from He et al. [16].

### 2.5. Tea Samples’ Sensory Evaluation

Green tea sensory evaluation: refer to our previous research for the specific scoring method used [16].

### 2.6. Volatile Compound Extraction and Identification in Tea Samples

The volatile compound content was determined by Headspace solid-phase microextraction (HS-SPME) combined with gas chromatography (GC) coupled with mass spectrometry (MS) (Thermo Fisher Scientific, Waltham, MA, USA) analysis according to the method described by Yang [17], with minor modifications. The specific information of the instrument and the parameters of the procedure are based on our previous research [16].

### 2.7. Data Processing and Statistical Analysis

The HS-SPME-GC-MS data preprocessing method for the green tea was identical to the method used in the previous study [16]. Excel 2016 and SPSS 26.0 were used to analyze the data. Origin 2022 and Prism 8.0 were used to draw figures.

## 3. Results

### 3.1. Green Tea’s Sensory Qualities in Relation to Various Light-Intensity Treatments

Green tea’s appearance was not evaluated in this study. The appearance score of green tea was set at 95.00 points. The results presented in Table 1 show that the change in light intensity affects the aroma and taste of green tea, but not the liquor color or the appearance of the waste leaves. Compared with the control group, ML treatment (94.00) significantly improved the aroma score of green tea; however, there was no significant difference observed between high-light-intensity (HL, 88.00) and low-light-intensity (LL 90.00) treatments. The results of the taste score showed that ML (95.67) has the highest score, while HL (88.33) has the lowest score. In addition, although the taste score for the ML treatment was significantly higher than that of the DARK treatment (91.33), there was no significant difference to that of the NL treatment (93.33).

### 3.2. Volatile Compound Production According to Different Light-Intensity Treatments

#### 3.2.1. Classification of Volatile Components

**Figure 2:**
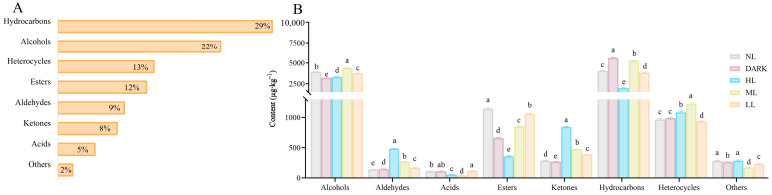
Analysis of volatile substances in green tea. (**A**) The proportion of volatile compounds classified according to functional groups. (**B**) The variation in content of volatile compounds between different treatment groups. Different letters in the same volatile categories indicate significant differences at *p* < 0.05 level.

Tea volatile compositions were comprehensively analyzed, and the results show that a total of ninety volatile compounds in the five tea samples of HL, ML, LL, NL, and DARK were detected (Table S1), in which eighty-eight volatiles were identified as hydrocarbons, alcohols, heterocycles, esters, aldehydes, ketones, and acids. The proportions of these chemical categories were 29%, 22%, 13%, 12%, 9%, 8%, and 5% in tea samples (Figure 2A).

Hydrocarbons, alcohols, heterocycles, and esters were the major volatiles in tea samples, accounting for 64% of the total amount of volatiles. Compared with the control group, the light treatment significantly increased alcohol, aldehydes, ketones, and heterocyclic compound contents in the green tea (Figure 2B). The major alcohols, such as linalool and linalool oxide (furanoid), were significantly increased under ML treatment during the spreading process, contributing an increase level of total alcohol content in the green tea. The heterocycle content, such as linalool oxide (pyranoid) and coumarin, increased significantly under ML treatment. In addition, HL treatment significantly increased aldehyde (benzaldehyde) and ketone (*trans*-*β*-Ionone) accumulation in the green tea, indicating that these compounds’ content increased with the light intensity. Four ketones were detected only under HL treatment. These volatile substances enriched the aroma components in the green tea products.

#### 3.2.2. Analysis of Total Content and VENE of Volatile Substances

**Figure 3:**
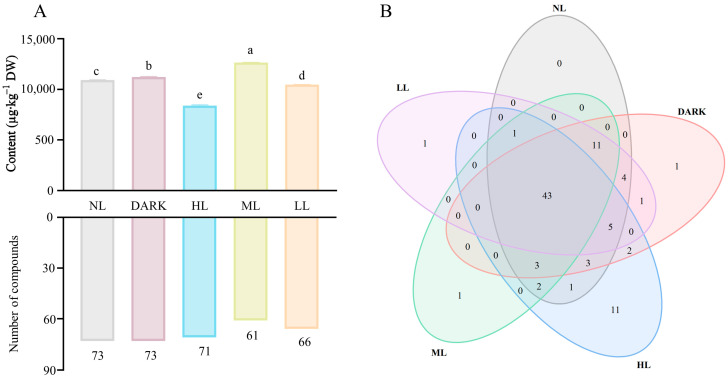
The distribution of volatile compounds in different treatment groups. (**A**) The content of total volatile compounds (**upper**, µg∙kg^−1^) and the number of volatile compounds (**lower**). Different letters in the same volatile categories indicate significant differences at *p* < 0.05 level. (**B**) Ninety distinct volatile chemicals are shown in the Venn diagram.

According to Figure 3A, seventy-three volatile compounds were detected in NL and DARK treatments, the highest number among the five treatment groups. Seventy-one, sixty-one, and sixty-six volatile compounds were detected in HL, ML, and LL treatments, respectively. Compared with the control groups, the total amount of volatile substances in the ML treatment (12,653.28 μg∙kg^−1^) was significantly increased. However, the total amounts of volatile components in HL (8412.07 μg∙kg^−1^) and LL (10,467.46 μg∙kg^−1^) treatments were significantly reduced, suggesting moderate intensity light was beneficial to increase the total volatile aroma of green tea during spreading. Interestingly, the green tea total aroma treated with HL was significantly lower than green tea treated with ML and LL treatments. However, aroma species of HL-treated green tea were 5- and 10-times more than LL and ML-treated green tea, respectively, indicating that HL treatment significantly decreased green tea total volatile compounds, but had no significant effect on the level of aroma.

The Venn diagram shows that forty-three common volatile compounds was detected in five treatments (Figure 3B). Three unique substances, hexanoic acid ethyl ester, butyrolactone, and (*Z*,*Z*)-3-hexenoic acid-3-hexenyl ester, were detected in DARK, ML, and LL treatments, respectively. However, no substances were detected in the NL treatment that were different from the other treatments. Eleven unique compounds were detected in the HL treatment. These results indicate that HL treatment may have affected the composition of volatile compounds in the green tea.

### 3.3. Green Tea Odor Profiles during Spreading under Different Light-Intensity Treatments

#### 3.3.1. Principal Component Analysis

**Figure 4:**
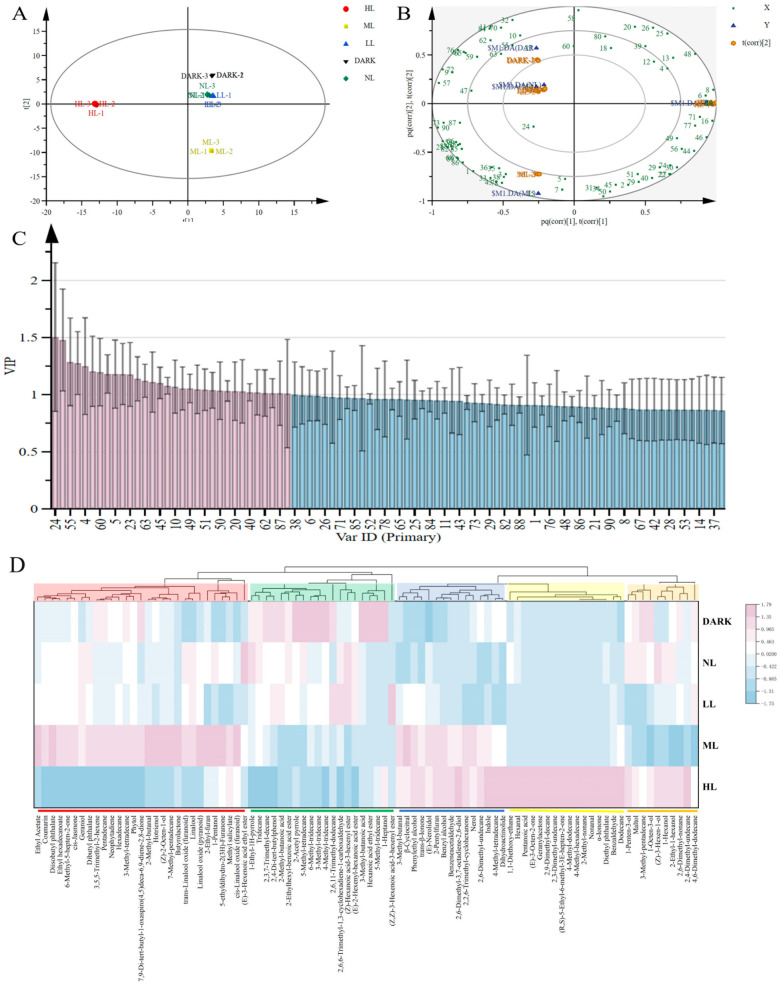
Distinctive volatile components of green tea in various conditions of light intensity. (**A**) Green tea treated under varying light-intensity settings shows a score plot of volatile chemicals derived from principal component analysis. (**B**) Biplot of discrimination analysis using orthogonal partial least squares for volatile chemicals in green tea treated under various light conditions. The serial numbers of the chemicals in Table S1 correspond to the numbers in the figure. (**C**) Significant variables in the projection (VIP) plot containing volatile chemicals found in green tea are orthogonal partial least squares discriminating analysis variables. Blue bars show 1 < VIP, and red bars show volatile chemicals with VIP > 1. (**D**) The depth of the color indicates the degree. Blank indicates a lack of enrichment. Cluster analysis using a heatmap. In the illustration, a volatile component is represented by each column and the treatment condition by each row. The abundance of information about the associated volatile components in the related green tea samples is represented by the color’s depth. Red denotes upregulation and blue denotes downregulation. The degree is indicated by the color’s depth. A blank represents a deficiency in enrichment.

To distinguish the differences between different light-intensity treatments, the principal component analysis (PCA) was conducted on volatile compounds. The five tea samples were well separated according to the light-intensity treatments. PC1 direction explained 50.8% of the maximum variation and PC2 explained the remaining 32.1%. As shown in Figure 4A, the five light treatments can be effectively distinguished. The difference between HL and other groups was greater than the distance between samples. ML treatment was in the lower-right quadrant and was distinguished from other groups. In the coordinate quadrant’s upper-right corner, LL, NL, and DARK treatments formed a cluster.

#### 3.3.2. Orthogonal Partial Least Squares Discriminant Analysis

To further explore the key volatile substances during the green tea spreading process under different light-intensity treatments, orthogonal partial least squares discriminant analysis (OPLS-DA) was performed. As shown in Figure 4B, samples under varying light intensities could be separated and predicted using the volatile compounds that were discovered as independent variables. Subsequently, the variable influence on the projection (VIP) parameter was used to select the metabolites, which exhibited significant contributions to volatile compounds under different treatments in the OPLS-DA model. The VIP is the variable weight value of OPLS-DA model variables, which can be used to measure the influence of the accumulation difference of each metabolite on the classification and discrimination of samples in each group and the explanatory power. VIP ≥ 1 is a common screening criterion for differential metabolites [18]. In the present study, a total of thirty-two differential volatile compounds with a VIP value larger than 1 were screened out (Figure 4C), indicating that they have an above-average influence on the differentiation of green tea with different light intensities. These include ten alcohols, three aldehydes, five esters, one ketone, one acid, seven hydrocarbons, and five heterocycles (Table S2). The results show that different light conditions have the greatest influence on alcohol in the green tea.

#### 3.3.3. Heatmap Cluster Analysis of Green Tea under Different Light-Intensity Treatments

Cluster analysis was used to explore the differences under different light-intensity conditions. The volatile compounds’ content data for the five treatment groups were normalized (Z-score) and then a cluster heat map was constructed. As shown in Figure 4D, row clustering shows that LL, DARK, and NL samples with a similar aroma are first clustered into one class, whereas HL and ML samples form their own category. The results shown in the clustering heatmap are the same as the PCA results.

In addition, column clustering showed that ninety kinds of volatile compounds were grouped into five major categories. The first category (marked in red in Figure 4D) included volatile compounds that were more associated with ML treatment, such as 2-methyl-butanal and (*Z*)-2-octen-1-ol. The second category (marked in green in Figure 4D) included volatile compounds that were highest in the DARK treatment, such as 2,6,11-Trimethyl-dodecane and 2-acetyl pyrrole. The third category (marked in blue in Figure 4D) included volatile compounds that were more associated with HL and ML treatments, such as (*E*)-nerolidol and benzyl alcohol. The fourth category (marked in yellow in Figure 4D) included volatile compounds that were highest in HL treatment, such as nonanal and α-lonone. The last category (marked in orange in Figure 4D) included volatile compounds that were lowest in ML treatment, such as 1-octen-3-ol and (*Z*)-3-hexen-1-ol.

#### 3.3.4. Validation of the Fourteen Important Differential Compounds under Different Light-Intensity Treatments

The ratio of the concentration to odor threshold (OAV) determined the odor characteristics of a volatile compound and was often used to analyze the aroma contribution of volatile compounds to tea odor, OAV > 1 indicating that the volatile compound had a certain effect [19].

In green tea treated with varying light intensities, fourteen major volatile chemicals were found when combined with OPLS-DA analysis and the OAV value (Table 2), including (*Z*)-3-hexen-1-ol, 5-ethyldihydro-2(3*H*)-furanone, 1-pentanol, methyl salicylate, 1-hexanol, 3-methyl-butanal, *trans*-linalool oxide (*furanoid*), nerol, *β*-cyclocitral, *trans*-*β*-ionone, 1-octen-3-ol, phenylethyl alcohol, linalool, and safranal. Among these compounds, linalool, 3-methyl-butanal, 1-octen-3-ol, and *trans*-*β*-ionone showed an OAV value > 10, indicating their substantial contributions to the overall aroma profile of the green tea.

**Figure 5:**
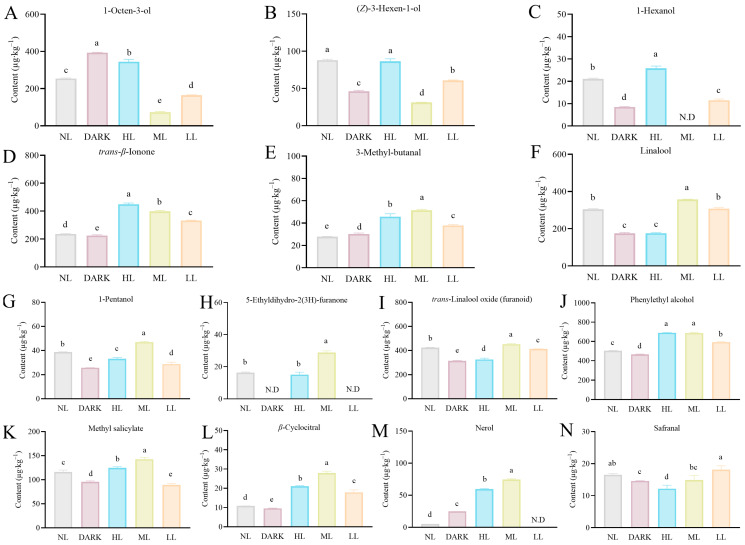
The impact of varying light-intensity levels on the concentrations of 14 significant differential volatile compounds that contribute to the distinctive green tea aroma. (**A**–**C**) Volatile substances that give green tea its grassy flavor. (**D**–**F**) Volatile substances that give green tea its chestnut-like aroma. (**G**–**N**) Volatile substances that give green tea its floral and fruit aroma. Values below the instrument detection limit are indicated by N.D. Different letters in the same volatile categories indicate significant differences at *p* < 0.05 level.

As shown in Figure 5A–C, the primary odorants responsible for the grassy smell of green tea are found to be 1-hexanol, 1-octen-3-ol, and (*Z*)-3-hexen-1-ol. ML treatment dramatically decreased the amount of these three substances in green tea, compared to the NL and DARK treatments. In addition, the content of 1-hexanol following ML treatment was even below the detection limit. The concentration of (*Z*)-3-hexen-1-ol, on the other hand, was the same following NL treatment, and was significantly higher in the HL treatment than in the NL treatment for 1-octen-3-ol and 1-hexanol. While the contents of 1-octen-3-ol, (*Z*)-3-hexen-1-ol, and 1-hexanol following the LL treatment were considerably higher than in the ML treatment, they were significantly lower following the NL treatment.

We discovered that the main odorants in green tea that produced it a chestnut-like scent were *trans*-*β*-ionone (violet, raspberry, and floral), 3-methyl-butanal, and linalool (Figure 5D–F). The *trans*-*β*-ionone content was significantly promoted under HL treatments and positively correlated with the light intensity. Under light treatment, 3-methyl-butanal content also increased dramatically, reaching its maximum value under ML treatment. Under ML treatment, the level of linalool was much higher; under HL treatment, it was significantly lower.

It has been determined that eight chemicals (Figure 5G–N) with fruity and flowery aromas play a significant role in the aroma of green tea. The contents of six volatile compounds, 1-pentanol (*fruity*), 5-ethyldihydro-2(3*H*)-furanone, trans-linalool oxide (*furanoid*), *β*-cyclocitral, methyl salicylate, and nerol, were the highest following ML treatment, and significantly higher than those following NL and DARK treatments. Compared with NL and DARK treatments, the light treatment significantly increased the content of phenylethyl alcohol (floral and rose-like) in the green tea. However, safranal (woody, spicy, medicinal, powdery, and herbal) content did not significantly increase following light treatment.

## 4. Discussion

The market value of tea depends on its quality, including color, freshness, strength, and aroma. Phenolic compounds play an important role in the tea’s color and taste, while volatile compounds are essential for tea odor and aroma [8]. There are many factors during tea cultivation, production, and processing affecting the tea’s flavor. Tea’s flavor consists of aroma and taste, which contain volatile compounds and non-volatile compounds. There are many criteria in tea quality evaluations, among which volatile aroma is one of the most important indexes [21]. Fresh tea contains various volatile compounds, and different processing methods affect the content and types of these compounds. In some instances, newly formed volatiles can also be beneficial for tea’s aroma. Most of green tea’s aroma comes from the fresh leaves or non-enzymatic reactions during manufacturing because the fixation process of green tea leads to most of the enzymes being inactivated [22].

Light is an important environmental factor affecting tea quality. During the spreading process of the fresh tea leaves, light regulates the activities of cellular metabolites and main enzymes, affecting the major quality components [23]. Recent developments and applications of light-emitting diodes provide opportunities for the targeted regulation of plant growth and plant nutrient accumulation to optimize yield and quality in a controlled environment [24]. In green tea, the main purpose of the spreading treatment is to evaporate water, regulate enzyme activity, and transform characteristic components of post-harvest tea [25]. Recently, artificial-light withering has become a research hotspot. Previous studies mainly focused on the exploration of light-quality conditions during tea spreading after harvesting [11,14,15]. For example, red and blue lights can promote black tea’s aroma and taste [26]. However, little has been published about the effects of light intensity on tea quality during spreading. In this study, the results of the sensory evaluation show that green tea spreading under the ML condition has the highest aroma score and is significantly higher than the other treatment groups. This was mainly reflected in the fact that ML significantly contributed to the chestnut-like aroma formation and inhibited the “raw grass” aroma in the green tea. We surmised that the ML condition accelerated the evaporation or conversion of boiling volatile compounds. Compared with HL and LL treatments, ML-treated green tea obtained a higher taste score, which could be related to the non-volatile substances in the tea leaves, and further studies are needed to prove this result.

Prior research has demonstrated that the production of alcohols, esters, and hydrocarbons during withering benefited from higher light intensity (red) [9]. Additionally, our earlier research revealed that, while HRL was detrimental to the creation of alcohols, esters, and hydrocarbons, an increase in red-light intensity was advantageous to the accumulation of alcohols, aldehydes, esters, and ketones in green tea [16]. In the present study, we found that ML favored the accumulation of alcohols and heterocycles, and HL favored ketone and aldehyde generation in green tea. In fresh tea, oxidative cracking and the decarboxylation of various fatty acids produced volatile fatty acid derivatives, which then produced short-chain volatiles of aldehyde and ketone moieties [27]. The OPLS-DA analysis showed that 18 of the 32 key volatile compounds were volatile fatty acid derivatives, suggesting that light treatment improved aroma synthesis in the fatty acid metabolic pathway of the tea leaves. Therefore, it is hypothesized that by raising the activity of enzymes involved in fatty acid metabolism, light treatment could encourage the breakdown of fatty acids. Prior research has demonstrated that light conditions are essential for terpene production and accumulation, potentially because light stimulates the expression of genes involved in the MEP pathway [28]. In this study, four key volatile terpenes were screened, including *trans*-linalool oxide (furanoid), nerol, *β*-cyclocitral, and linalool. The contents of these four substances were significantly increased under the ML condition and decreased under HL and LL conditions. Compared with the dark treatment, LL treatment significantly increased the contents of *β*-cyclocitral and linalool in green tea. It may be that the increased light intensity affects the activity of terpene synthetase in the terpene synthesis pathway. The concentrations of *β*-cyclocitral and linalool in green tea were significantly higher after the LL treatment than after the dark treatment. This could be because the increased light intensity affects the activity of terpene synthetase in the terpene synthesis pathway. Because the synthesis of terpenes in tea leaves was unaffected by the lower light intensity, the contents of *trans*-linalool oxide (furanoid) and nerol in the LL treatment group were significantly lower than those in the NL treatment group. This study revealed that light intensity affected the terpene content in green tea.

Green tea contains a variety of volatiles (up to 200), but only 30 volatiles were identified to be responsible for green tea’s characteristic aroma [29,30,31]. The chestnut scent is a common aroma characteristic of some well-known green teas in China, and it is also a crucial indicator of the quality of tea [32]. The aroma of green tea containing “raw grass” can seriously affect its flavor, and (*Z*)-3-hexene-1-ol may be the main component causing this smell [33]. By calculating the OAV value, we were able to identify fourteen essential aroma components that made a substantial contribution to the aroma quality of the green tea in our study. Linalool and 3-methyl-butana as volatile compounds representing the green tea chestnut-like aroma were significantly increased under ML conditions. Under HL treatment, there was a considerable upregulation of *trans*-β-ionone, a volatile molecule with a scent similar to chestnut-like green tea. The concentration of this chemical showed a positive correlation with the intensity of light. (*Z*)-3-hexen-1-ol, 1-octen-3-ol and 1-hexanol are volatile compounds that represent the scent of grass, and their content increased significantly under the HL condition but decreased significantly under the ML condition. This could be because the ML condition sped up the creation of high-boiling volatile compounds with a chestnut flavor by encouraging the quick volatilization or transformation of low-boiling volatile compounds with a grass-like flavor. Floral aroma is a characteristic of high-quality green tea. In this study, the contents of substances representing floral aroma, 1-pentanol, 5-ethyldihydro-2(3*H*)-furanone, *trans*-linalool oxide (furanoid), methyl salicylate, *β*-cyclocitral, and nerol were significantly increased under the ML condition [34,35,36]. The results show that ML improved the aroma quality of green tea by enhancing the chestnut aroma and reducing the grass aroma.

In conclusion, using a compound light source with a medium light intensity throughout the spreading phase can increase the quality of the green tea scent. The results of this investigation help us to comprehend how aromas are formed in green tea when it spreads under various light conditions.

## 5. Conclusions

HS-SPME-GC-MS was used to systematically study the effects of different light intensities on the volatile metabolites in green tea. Ninety non-volatile compounds were identified, including twenty-six hydrocarbons, twenty alcohols, twelve heterocycles, eleven esters, eight aldehydes, seven ketones, and four acids; the results reveal the changes in volatile metabolites of green tea under different light intensities. Fourteen of the most important key aroma compounds in green tea were selected from ninety volatile compounds. Among these, the ML condition significantly increased the chestnut-like aroma and decreased the “raw grass” odor in green tea. Thus, the present study demonstrates that the aroma quality of green tea can be improved by changing the light intensity. Our study provided a comprehensive and systematic theoretical basis for the application of red-light and medium-intensity composite-light sources as the tea processing quality control in the tea withering process, and provided the targeted technical guidance for green tea production.

## Figures and Tables

**Table 1:** The findings of the green tea samples’ sensory evaluation under various light conditions.

Light Condition	Aroma	Taste	Liquor Color	Appearance of Waste Leaves	Comprehensive Sensory Evaluation
HL	88.00 ± 2.00 b	88.33 ± 2.08 c	89.00 ± 4.00 a	84.67 ± 4.04 a	89.62 ± 1.11 c
ML	94.00 ± 1.73 a	95.67 ± 0.58 a	94.00 ± 3.00 a	90.33 ± 4.51 a	94.38 ± 0.78 a
LL	90.00 ± 1.00 b	92.33 ± 1.53 b	92.33 ± 3.06 a	89.67 ± 2.08 a	92.15 ± 0.57 b
DARK	90.00 ± 0.00 b	91.33 ± 1.15 b	92.00 ± 1.00 a	89.33 ± 0.58 a	91.78 ± 0.31 b
NL	90.00 ± 2.00 b	93.33 ± 1.53 ab	92.00 ± 2.00 a	89.33 ± 1.15 a	92.38 ± 0.15 b

Notes: one-way analysis of variance (ANOVA) was performed, and different letters in the same column indicate a significant difference between light conditions (*p* < 0.05).

**Table 2:** Important volatile substances with an OAV > 1.

Compound Name	Odor Characteristic	OTs (µg∙kg^−1^)	OAV				
			HL	ML	LL	DARK	NL
(*Z*)-3-Hexen-1-ol	Green, leafy, grassy	70	1.23 ± 0.05 a	0.45 ± 0.01 d	0.87 ± 0.01 b	1.26 ± 0.02 a	0.66 ± 0.02 c
5-ethyldihydro-2(3*H*)-Furanone	Caramel, nutty, roasted, sweet, creamy	9.7	1.56 ± 0.16 b	2.97 ± 0.11 a	N.D	1.69 ± 0.04 b	N.D
1-Pentanol	Fruity	5	6.6 ± 0.23 c	9.39 ± 0.11 a	5.74 ± 0.33 d	7.74 ± 0.04 b	5.12 ± 0.01 e
Methyl salicylate	Minty, wintergreen-like	40	3.11 ± 0.07 b	3.57 ± 0.09 a	2.23 ± 0.07 c	2.9 ± 0.1 b	2.39 ± 0.05 c
1-Hexanol	Green, grassy	5.6	4.62 ± 0.17 a	N.D	2.07 ± 0.1 c	3.77 ± 0.06 b	1.52 ± 0.04 d
3-Methyl-butanal	Apple-like and chocolate-like flavors under high dilution	0.2	228.81 ± 13.23 b	257.14 ± 4.57 a	189.91 ± 4.27 c	138.98 ± 1.16 d	151.57 ± 4.2 d
*trans*-Linalool oxide (*furanoid*)	Sweet, floral, creamy	190	1.72 ± 0.05 c	2.38 ± 0.02 a	2.17 ± 0.01 b	2.23 ± 0.01 b	1.65 ± 0.02 c
Nerol	Fresh, citrus, floral, green, sweet, lemon-like	49	1.22 ± 0.02 b	1.52 ± 0.02 a	N.D	0.1 ± 0 d	0.51 ± 0 c
*β*-Cyclocitral	Herbal, clean, rose-like, fruity	3	7.02 ± 0.1 b	9.3 ± 0.32 a	5.98 ± 0.41 c	3.63 ± 0.01 d	3.19 ± 0.05 d
*trans*-*β*-Ionone	Violet, raspberry, floral	0.007	64,204.26 ± 1270.98 a	56,976.93 ± 916.74 b	47,613 ± 360.76 c	33,787.68 ± 418.39 d	32,198.97 ± 686.29 d
1-Octen-3-ol	Earthy, green, oily, vegetable-like, fungal	1	344.92 ± 12.82 b	73.29 ± 3.9 e	164.47 ± 2.73 d	254.03 ± 3.24 c	393.34 ± 2.37 a
Phenylethyl alcohol	Floral, rose-like	390	1.77 ± 0.01 a	1.76 ± 0.02 a	1.52 ± 0.01 b	1.29 ± 0.01 c	1.19 ± 0.01 d
Linalool	Floral, sweet, grape-like, woody	0.22	798.65 ± 15.82 c	1624.81 ± 5.67 a	1401.15 ± 20.29 b	1384.34 ± 14.01 b	796.2 ± 19.8 c
Safranal	Woody, spicy, medicinal, powdery, herbal	3	4.06 ± 0.36 c	4.95 ± 0.48 bc	6.04 ± 0.4 a	5.5 ± 0.13 ab	4.87 ± 0.02 bc

Notes: odor threshold (OT) reviewed in Leffingwell & Associates Threshold Value Database (https://www.leffingwell.com/, accessed on 5 May 2023, and other literature [20]. Values below the instrument detection limit are indicated with N.D. Different lowercase letters in a row indicate a significant difference between fixation treatments (*p* < 0.05).

## Data Availability

The original contributions presented in the study are included in the article. Further inquiries can be directed to the corresponding authors.

## Supplementary Materials

The following supporting information can be downloaded at: https://www.mdpi.com/article/10.3390/foods13152349/s1, Table S1: Volatile compounds were detected by HS-SPME-GC-MS in green tea; Table S2: Volatile components screened according to VIP > 1 and *p* < 0.05 in green tea.
